# Targeting of Regulators as a Promising Approach in the Search for Novel Antimicrobial Agents

**DOI:** 10.3390/microorganisms10010185

**Published:** 2022-01-15

**Authors:** Davide Roncarati, Vincenzo Scarlato, Andrea Vannini

**Affiliations:** 1 Department of Pharmacy and Biotechnology (FaBiT), University of Bologna, 40126 Bologna, Italy; 2 Department of Experimental, Diagnostic and Specialty Medicine, University of Bologna, 40126 Bologna, Italy

**Keywords:** bacterial pathogens, gene expression, virulence factors regulation, antibiotic resistance, new antibacterial targets

## Abstract

Since the discovery of penicillin in the first half of the last century, antibiotics have become the pillars of modern medicine for fighting bacterial infections. However, pathogens resistant to antibiotic treatment have increased in recent decades, and efforts to discover new antibiotics have decreased. As a result, it is becoming increasingly difficult to treat bacterial infections successfully, and we look forward to more significant efforts from both governments and the scientific community to research new antibacterial drugs. This perspective article highlights the high potential of bacterial transcriptional and posttranscriptional regulators as targets for developing new drugs. We highlight some recent advances in the search for new compounds that inhibit their biological activity and, as such, appear very promising for treating bacterial infections.

## 1. Introduction

The ability of bacteria to gauge surroundings and modulate gene expression accordingly represents a crucial feature for their survival. Also, bacterial pathogens sense and respond to environmental signals to establish a successful infection, survive, and replicate within the host. To this end, coordinated regulation of metabolic and virulence gene expression is required for pathogenic bacteria to compete for nutrients within the resident host and escape the immune system. Such a response is achieved mainly by employing DNA-binding regulatory proteins controlling the initiation of gene transcription and small regulatory RNAs acting at the post-transcriptional level.

Bacteria typically enroll an arsenal of transcriptional regulators to orchestrate gene transcription in response to environmental stimuli. Once an external signal is perceived, it is transduced into the cell to harmonize specific molecular mechanisms controlling the transcription of genes that code for proteins capable of assisting the cell in adapting to the new condition. Dedicated regulatory proteins can mediate transcriptional responses under either positive or negative control. Positive regulation of transcription exploits distinct alternative sigma factors to redirect the RNA polymerase enzyme to a subset of specific gene promoters, as well as by transcriptional activators or their combination. In contrast, transcriptional repressors mediate negative regulation. Moreover, these two opposite strategies coexist in some microorganisms, establishing complex regulatory networks.

A paradigm of such systems is the well-studied heat-shock response of several bacterial pathogens, a crucial protective mechanism for bacterial survival and adaptation to hostile environmental conditions. A recent overview of the foremost mechanisms adopted by different pathogenic bacteria to cope with the heat-shock response is available [1], along with the identification of molecules able to bind to a variety of bacterial transcriptional regulators to block their function, and thus with the potential for the development of new antibiotics [2].

In addition to regulatory proteins, bacteria adopt a broad class of small RNAs (sRNAs) to respond to environmental changes. Growing studies on bacterial regulatory processes establish that sRNAs generally modulate their target gene expression at the post-transcriptional level. These can act both as positive and negative regulators. The basis of their mechanism of action resides in the acquisition of secondary structure with unpaired regions, which serve as a pairing motif to select their targeted mRNA. Often, sRNA activity depends on the interaction with a small RNA binding protein that assists folding and interaction. So far, Hfq, CsrA, and ProQ represent the best-characterized examples of RNA chaperones involved in sRNA-mediated regulation. An overview of sRNA’s main features and roles in controlling gene expression in pathogenic bacteria is presented in a review published by Caldelari and co-workers and in references therein [3].

Deep comprehension of regulatory proteins and sRNA mechanisms of action is a prerequisite for developing new antibacterial drugs to treat bacterial infection in conjunction with or alternative to antibiotic treatment. Nowadays, it is well established that antibiotics profoundly combat infectious diseases and mortality. However, pathogens develop defense strategies against them as we use the drugs, which result in less or no effectiveness. Therefore, new approaches to treat infectious diseases that reduce resistance are needed. Accordingly, increasing efforts have been put forward in searching for new bioactive compounds able to inhibit the growth of pathogens by acting specifically on new molecular targets for novel drug discovery. As a result, many transcriptional regulators have been considered and deeply studied as valid drug targets with promising results. The two-component systems (TCS) have received increasing attention over time among the regulatory proteins, and Hirakawa and colleagues have reviewed recent progress on natural and synthetic compounds [4]. Here, we briefly summarize progress and discuss future perspectives on developing new antimicrobial therapeutic strategies.

## 2. Transcriptional Regulators

In prokaryotes, regulation of transcription plays a pivotal role in the modulation of gene expression in response to environmental variations and growth. Transcription of genes into messenger RNAs (mRNAs) occurs through the activity of the DNA-dependent RNA polymerase (RNApol), which interacts with specific DNA sequences, i.e., the promoters, and copies the downstream genes into mRNAs. Promoter recognition by RNApol, transcription initiation, and elongation constitute the principal events of transcription regulation, and their modulation determines the expression of the gene(s) downstream the promoter. Hence, the first layer of transcription regulation consists of the interplay between the RNApol and the promoter sequences and relies on the intrinsic features of these two elements. The second layer of regulation consists of the transcription regulators (TRs), regulatory proteins that alter RNApol-promoter interactions and modify gene expression [5].

**Figure 1:**
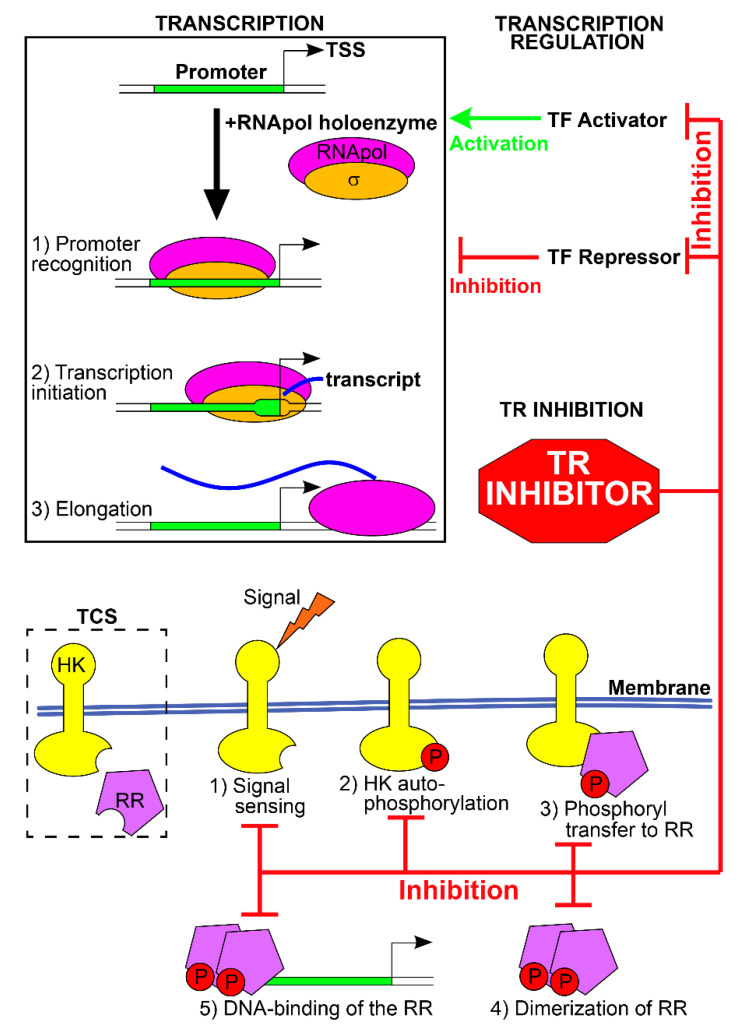
Transcription regulation and TRs as new antibacterial targets. Transcription regulation concerns the key events leading to transcription: promoter recognition by RNApol, core promoter melting and transcription initiation, and elongation (top left box). TRs are activators or repressors, and positively (green arrow) or negatively (red hammerhead) modulate these events, causing gene expression variations. Compounds targeting the TFs and inhibiting their function interfere with the expression of the TR regulon. TCSs typically consist of an HK and a RR (schematized in the broken-line box). Upon signal sensing, HK undergoes autophosphorylation, the phosphate group is transferred to the RR, which dimerizes and binds the DNA to regulate transcription (steps 1–5). Compounds targeting TCSs can inhibit the various stages of this process.

The first layer of regulation involves a specific subunit of the RNApol holoenzyme, the σ factor, which is essential for recognizing specific DNA sequences within the promoters (the core promoter) and for transcription initiation. Bacteria can produce different σ factors, and each of them recognizes a specific DNA sequence. Hence, the expression of a particular σ factor enables the transcription of the genes that harbor the σ-target sequence. The promoters of most of the genes expressed under normal growth conditions are recognized and bound by the primary housekeeping σ factor (σ^70^ for *Escherichia coli*). In contrast, alternative σ factors are expressed in response to environmental variations (heat shock, nitrogen or iron starvation), in specific points of the growth phase (stationary phase and sporulation), to coordinate the expression of the proteins involved in a cellular appendage (flagellum, pili, secretion system), in a metabolic pathway or virulence [6]. Changing the RNApol-bound σ factor allows the simultaneous expression of multiple and related genes to meet the temporary needs of the cell (Figure 1).

The second layer of regulation involves the TRs, which bind other specific nucleotide sequences of the promoters and activate or inhibit transcription. In prokaryotes, many TRs act as repressors of transcription, blocking the activity of the RNApol. To this aim, different mechanisms are employed: TRs can (i) occupy the core promoter and sterically hinder the binding of RNApol holoenzyme, (ii) block promoter melting and transcription initiation, (iii) bind DNA sequences downstream the promoter blocking transcription elongation, (iv) form locked DNA loops that block promoter recognition, transcription initiation or elongation, or (v) inactivate activators of transcription [5]. Other TRs acts as activators of transcription, supporting the recruitment of RNApol on the promoter, enabling transcription initiation, or inactivating the repressor TRs. Transcription regulators are typically homodimers, each formed by two distinct domains: one is the DNA-binding domain and recognizes the TR-specific DNA sequences. The other is the regulatory domain and acts as a sensor of the environmental signals. In the presence of the effector ligands or chemical modifications, the regulatory domain activates or inhibits the DNA-binding domain, switching on or off the TR and modulating transcription. Furthermore, transcription of TF-coding genes is often under autogenous control, i.e., the regulator controls its expression. The two-component transduction systems (TCS) constitute an important class of TR in which the sensor and the DNA binding elements are featured in 2 distinct proteins [7]. In presence of the specific environmental signal, the sensor histidine kinase (HK) activates itself by autophosphorylation, and then transfers the phosphoryl group to the cognate effector response regulator (RR), switching on or off its DNA-binding function and, consequently, its activity as TR. TRs recognize and bind a specific nucleotide sequence which often occurs at several promoters, granting the simultaneous regulation of multiple genes by the TR (the regulon of the TR) in response to the environmental signal. Moreover, bacterial genes are often organized into operons, in which a single promoter drives the expression of a cluster of downstream genes. Transcription regulation of the promoter usually co-regulates the entire cluster, granting the simultaneous regulation of many genes. Promoters can be targeted by multiple TRs, each regulating the transcription in response to a specific environmental signal (Figure 1). Lastly, the promoter of TR is often regulated by other TRs, forming complex regulatory networks in which the expression of a TR and of its regulon is enhanced or dampened by other TRs in response to multiple environmental signals [8].

### 2.1. TRs as New Targets for Antibacterial Therapies

Some TRs are fundamental for the survival of a specific bacterium, i.e., it is not possible to obtain mutant strains lacking these TRs, likely because they positively or negatively control the expression of genes with pivotal functions in the cell. On the other side, some TRs are dispensable for the in vitro viability of the bacteria and control genes for the survival in particular environmental conditions (acidic medium, starvation of a nutrient, heat-shock), for the expression of virulence factors, or host invasion. Essential TRs are ideal targets for the development of new antibiotics. In fact, the inactivation of TRs alters the expression of fundamental genes for cell viability, TRs have no counterpart in humans and are specific for a particular bacterial strain or class, and they are usually small soluble proteins that are easy to obtain and study for drug discovery and validation [2]. Specifically, the ease of handling makes the TRs suitable for different approaches: in silico (structure-based drug design using computational tools on resolved structures of TRs), in vitro (drug-TR binding assay, co-structural analyses, TR inhibition tests), and in vivo (bacteriostatic and bactericidal tests, TR inhibition tests in autologous or heterologous reporter systems). The inhibitory drugs could interfere with different functions of the TRs: signal perception, protein dimerization/oligomerization, DNA-binding activity (Figure 1) [4]. Non-essential TRs can also be attractive targets for drug discovery because their inactivation hampers the fitness in vivo and pathogenesis, with the outcome of preventing host colonization, reducing virulence, or weakening the evasion mechanisms from the host defenses and from conventional antibiotic therapies [9]. Targeting only virulence and pathogenesis, not cell viability, could reduce the selective pressure for the emergence of new antibiotic-resistant strains and likely has little to no detrimental effects on the normal microbiota [10]. To date, several studies have investigated the TRs of clinically relevant bacteria as targets for novel antibiotic or bacteriostatic compounds. In the following paragraphs, we provide some examples summarized in Table 1. The examples shown are only representative of some bacterial systems. Those discussed below have been chosen to represent different inhibition signals and are not a complete and exhaustive review of the various biological systems available in the literature.

### 2.2. Signal Sensing—ComD (Streptococcus pneumoniae)

*Streptococcus pneumoniae* is a leading cause of pneumonia, meningitis, and sepsis. In one-third of infections, bacteria are resistant to one or more clinically relevant antibiotics because *S. pneumoniae* can easily acquire antibiotic resistance genes by horizontal transfer when it enters the competent state. The competence-stimulating peptide (CSP) is a small molecule secreted by the bacterium, and it is employed as a quorum-sensing signal. When its concentration reaches a threshold, CSP is sensed by the ComD/ComE TCS, which activates and triggers a regulatory cascade, leading to the competent state and the expression of virulence factors. Dominant-negative analogues of CSP were tested for their ability to compete for binding to the sensor ComD (HK) without triggering the expression of factors for competence and virulence. Some molecules dampened the expression of ComD/ComE-regulated genes and reduced mouse mortality during lung infection by *S. pneumoniae* [49,50].

### 2.3. Autophosphorylation of HK—QseC (Many Bacteria)

QseC is an HK present in dozens of Gram-negative bacterial pathogens, including *Salmonella enterica*, *Haemophilus influenzae*, *E. coli* (enterohemorrhagic and uropathogenic strains), and *Aeromonas hydrophila*. When QseC senses the host stress hormones epinephrine and norepinephrine, or the bacterial autoinducer-3, it undergoes autophosphorylation and activates QseB, QseF, and KdpE RRs. In *E. coli*, these RRs control the expression of flagella and motility genes (QseB), Shiga toxin (QseF), and other virulence factors (KdpE). A screening identified the compound LED209 as a specific inhibitor of QseC [44], which allosterically modifies the HK and prevents its autophosphorylation [45]. In vitro treatments with LED209 decreased the expression of QseC-dependent virulence factors in many bacterial strains without affecting bacterial growth. Administration of LED209 during murine infection by *S. typhimurium* or *Francisella tularensis* suppressed the pathogenicity of these bacteria [45].

### 2.4. Phosphoryl Transfer to RR—BasR, CreB (E. coli)

BasS/BasR and CreC/CreB of *E. coli* are TCSs that increase the survival of the bacterium in presence of high amounts of ferric ions (BasS/BasR) or induce the expression of intermediary metabolic enzymes when cells are cultivated in minimal media (CreC/CreB). Lactoferricin B is a pepsin-digested peptide of bovine lactoferrin. This compound can penetrate the bacterial membrane and deactivate both TCSs, by selective binding of the RR receiver domain and inhibition of phosphoryl transfer from the HKs to the RRs. In vivo, Lactoferricin B reduced bacterial growth in the presence of excessive ferric ions and minimal medium conditions [37].

### 2.5. DNA Binding—HsrA (Also Known as HP1043, Helicobacter pylori)

*Helicobacter pylori* colonize the human stomach, and its infection causes peptic ulceration, gastric adenocarcinoma, and MALT lymphoma. Most, or all, gastric cancers are accountable to *H. pylori* infection. HsrA, also referred to as HP1043, is a conserved and essential TR that functions as an activator of transcription for a plethora of housekeeping and redox genes [53,54]. This regulator is a RR of a TCS, but it is an orphan of its cognate HK, and it performs its function without the need for activation by phosphorylation. Structurally, the TR comprises a dimerization domain and a DNA-binding (transactivation) domain that recognizes a bipartite consensus sequence. A screening of 1120 FDA-approved drugs was performed to identify HsrA binders: 7 natural flavonoids interacted with the amino acids involved in forming the helix-turn-helix DNA-binding motif and inhibited the in vitro DNA-binding activity of HsrA. Four of them showed bactericidal activity against *H. pylori* [20].

### 2.6. Dimerization—WalK/WalR (Multiple Microorganisms)

WalK/WalR is an essential TCS of some Gram-positive bacteria (including *Bacillus subtilis*, *S. pneumoniae*, *Enterococcus faecalis*, and *S. aureus*), which regulates cell wall metabolism and membrane composition in response to cellular signals. To find inhibitors of WalK/WalR, a chemical library was screened, and a compound was found that specifically inhibited the dimerization of the HK upon activation. In vitro, the compound showed antibacterial activity against methicillin-resistant *S. aureus* (MRSA) and vancomycin-resistant *E. faecalis* (VRE) [31]. Inhibition of dimerization can also occur at the level of RR [29]. Other compounds inhibit WalK/WalR TCS with different mechanisms: blocking the autophosphorylation of HK [30,32] or the phosphoryl transfer to RR [29].

### 2.7. Unknown—VirF (Shigella flexneri)

*Shigella flexneri* is a human pathogen that invades, inflames, and kills the colon epithelia, resulting in watery diarrhea and leading to more than 1 million deaths each year, the majority of which are children. VirF is a non-essential TR that functions as the master transcriptional activator of the virulence factors required for pathogenesis. At the same time, its inactivation does not impair the viability of the cell. Transcription of VirF is thermoregulated and occurs only at the shift from environmental to host temperature. Once that happens, VirF triggers a regulatory cascade that involves the transcription of *icsA* for cell invasion and of *virB*, which in turn activates the expression of other virulence factors required for pathogenesis [55]. Employing a *virB* promoter-*lacZ* reporter specifically activated by VirF, different libraries of chemical and natural compounds were tested for their ability to interfere with the activity of VirF without affecting the viability of the bacterial and host cells [22]. Six compounds met the criteria, and four reduced host cell invasion by inactivating the VirF-dependent cascade.

## 3. Posttranscriptional Regulators (sRNA)

In prokaryotic organisms, gene expression is not only controlled at the level of transcription initiation by the action of DNA-binding proteins. In fact, over the past few decades, extensive research efforts have led to the identification of new classes of RNA molecules (sRNA), often small-sized and almost always non-coding, which appear as major players in gene regulation through disparate mechanisms of action [56]. Following the first discoveries of sRNAs in *E. coli* (MicF and Spot42 [57,58]) and *S. aureus* (RNAI and RNAIII [59,60]), the number of reported sRNAs has exploded in the last decade with the emergence of high-throughput technologies like high-density tiling microarrays and RNA-sequencing, which allow an unbiased and deep analysis of bacterial transcriptomes. Nowadays, sRNAs have been discovered in virtually all bacterial organisms. The definition of their regulatory functions and mechanisms of action is progressing fast, unravelling the high complexity of their interactomes. Moreover, modern high-throughput methodologies that profile all sRNA-RNA interactions in the cell have allowed understanding the regulatory complexity controlled by sRNAs, shedding light on the collaborative work exerted by sRNA and DNA-binding transcriptional regulators.

### 3.1. sRNAs-Mediated Mechanisms of Regulation

**Figure 2:**
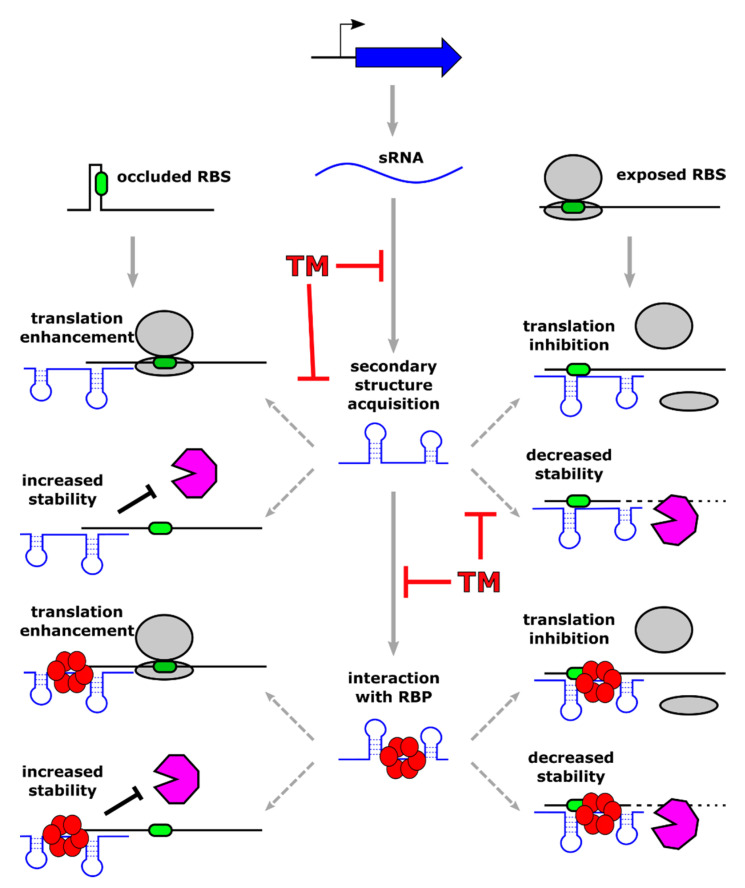
Schematic representation of the principal mechanisms of action used by base-pairing regulatory sRNAs and possible targeting strategies through antisense short oligonucleotides. A regulatory sRNA binds one or more target transcripts with or without the assistance of an RNA-binding protein (shown as red circles forming a hexamer associated to the sRNA). This interaction results in either positive or negative modulation of gene expression. Left side of the figure: positive regulation occurs when sRNA binding leads to increased ribosome binding site (RBS, represented by a green oval) accessibility and/or protection of the transcript from RNase (depicted by a pink polygon) processing. Right side of the figure: negative regulation of gene expression takes place when sRNA interaction with the target transcript occludes the RBS, preventing ribosome loading (ribosomes are represented by two grey ovals), and/or promoting RNase-mediated processing. Novel sRNA-targeting molecules (indicated with the symbol “TM” in the figure) could act in principle in several ways, affecting the different steps of sRNA-mediated regulation.

Typically, sRNAs act at the posttranscriptional level, influencing the stability or the translation efficiency of the regulatory targets. These latter include most often other RNAs that are recognized through base-pairing between complementary regions belonging to the two interacting molecules. More in detail, when the sRNA-target RNA interaction takes place in the 5′ region of a coding transcript, it can down-regulate (or less frequently enhance) mRNA translation. In addition, the sRNA-target RNA duplex that forms can lead to modulation of target stability through its exposure or protection from active RNase-mediated degradation (Figure 2).

Different sRNA subclasses show the varying extent of complementarity with target RNA. Some sRNAs share an extended stretch of perfect complementarity (also known as antisense or cis-encoded sRNAs), while others pair to their target molecules through few and often non-consecutive complementary bases (trans-encoded sRNAs). In this latter case, sRNAs seek the assistance of RNA chaperones, which favor sRNA-target RNA interaction and stabilize the regulatory molecule in the cytoplasm [61]. At present, the best-characterized RNA chaperones are Hfq and CsrA [62,63], while only in recent years another RNA-binding protein named ProQ has been identified, showing a broad spectrum of RNA ligands. The determinants that drive specific RNA ligands recognition by RNA chaperones are still ill-defined. However, recent studies highlight the importance of both short stretches of sequence conservation and RNA secondary structures [64]. Besides the widespread modes of action modulating translation and/or stability described above, sRNAs can modulate the expression of target genes employing a plethora of diverse mechanisms, including the control of transcription termination (recently reviewed by Bossi and co-workers [65]). Moreover, some sRNAs play their regulatory role in binding target proteins rather than other RNA molecules. These sRNAs are able to inhibit the activity of the target protein by mimicking the structures of their target mRNA, although the examples characterized so far are just a handful [66].

The expression of non-coding regulatory RNAs is responsive to specific environmental variations, including heat-shock, oxidative stress, metal availability, specific carbon source, transition to stationary phase, and several other challenges. Similarly to what happens for several DNA-binding regulators that control transcription of many genes in response to a specific signal, many sRNAs can bind to multiple targets and act as regulatory “hubs”, coordinating the expression of a large number of genes involved in a wide range of cellular processes [67,68].

### 3.2. Examples of Regulatory sRNAs Controlling Virulence and Pathogenesis

Interestingly, in bacterial pathogens, sRNAs are required for directly or indirectly regulating virulence and pathogenesis. The sRNA RNAIII of *S. aureus* represents a clear example of a single riboregulator coordinating the transition from colonization to infection. Specifically, RNAIII operates a direct negative regulatory effect on several genes (*coa*, *spa*, *sbi*, *sa1000*) involved in adhesion and immune evasion by inhibiting their translation and reducing transcripts’ stability [69]. Through the same mechanisms of action, RNAIII downregulates the expression of the transcriptional regulator Rot, thereby indirectly impacting several Rot-controlled genes coding for exotoxins. At the same time, RNAIII promotes the translation and stabilization of the transcripts of the alpha-toxin gene *hla* and of the MgrA regulator, which controls the expression of surface proteins and activates the synthesis of the capsule [70]. In the Gram-negative human pathogen *H. pylori*, the CncR1 sRNA oppositely modulates bacterial motility and adhesion to host cells. This sRNA is regulated by the essential transcriptional regulator HP1043 and is expressed within the 5′UTR of the *cagP* gene, which belongs to the *cag*-pathogenicity island, a major virulence determinant of *H. pylori*, encoding a type IV secretion system [71]. CncR1 inactivation leads to downregulation of genes related to host-pathogen interaction and upregulation of genes involved in regulation and assembly of flagellar apparatus. Among these motility-related genes, it has been shown that CncR1 directly targets multiple regions of *fliK*, a gene coding for a flagellar hook-length control protein. In addition, the experimental demonstration that CncR1 is necessary for *H. pylori* adhesion to host cell portrays this sRNA as a major regulator of motility and adhesion phenotypes of this important gastric pathogen [72]. Other recent studies identified a sRNA in *H. pylori* controlling the major virulence factors, defining it as a master regulator of the pathogen involved in the process of colonization of its host. The sequence of this sRNA named NikS is strongly conserved among *H. pylori* strains and is transcriptionally controlled by the NikR transcriptional regulator in response to nickel [73]. Two different studies identified a total of 11 direct target transcripts for NikS, coding for virulence determinants and outer membrane proteins, some being potential adhesins [74,75]. Of note, it has been shown that NikS targets the mRNA encoding the oncoprotein CagA and the vacuolating cytotoxin VacA, the two major virulence factors of *H. pylori*. Bacterial sRNAs have been implicated not only in the control of pathogen’s virulence traits but also in the modulation of host-pathogen communication. Several pathogens, including *S. aureus*, *H. pylori*, and *Pseudomonas aeruginosa*, can deliver some of their sRNAs into host cells from outer membrane vesicles [76,77,78]. Once inside the host, these bacterial riboregulators could intervene in the control of the host immune response [79].

### 3.3. Examples of Regulatory sRNAs Involved in Antibiotic Response and Resistance

Interestingly, many reports showed that, in several cases, sRNAs are induced following antibiotic treatment and take part in antimicrobial responses and resistance. In *E. coli*, the pleiotropic RNA regulator RhyB, a well-studied sRNA induced upon iron-starvation and involved in the control of iron homeostasis [80], plays a role in antibiotics resistance against four classes of molecules (aminoglycosides, β-lactams, fluoroquinolones, and tetracyclines) and *rhyB* mutant cells result more susceptible to antibiotic treatment as a consequence of dysregulation of respiratory complexes. In many other examples, sRNA regulation impacts antibiotic susceptibility through direct interaction and regulation of transcripts coding for proteins involved in cell-wall synthesis, antibiotic influx or efflux, and the overall composition of the bacterial envelope. Prominent examples of sRNA involved in controlling antibiotic resistance have been described in medically relevant pathogens like Methicillin-Resistant *S. aureus* (MRSA), *P. aeruginosa*, *Acinetobacter baumannii*, and *Neisseria gonorrhoeae* (extensively reviewed by Mediati and co-workers [81]).

Finally, in a recent study, it has been reported that the sRNA SprF1, previously characterized as an antitoxin RNA repressing the expression of the cytolytic peptide and virulence factor SprG1 of *S. aureus* [82], modulates ribosomes activity during translation and promotes the formation of persister cells, a subpopulation of dormant bacteria which are transiently resistant to different antibiotics [83].

### 3.4. Regulatory sRNAs as Targets for Novel Antibacterial Molecules

Given the crucial importance of sRNA-mediated regulation that has been observed in virtually all pathogenic bacteria, these riboregulators represent an appealing target for the design of novel antimicrobial approaches. Antimicrobial molecules that are able to target bacterial RNA already exist: several available antibiotics inhibit translation through direct binding to bacterial rRNAs (for example chloramphenicol and tetracyclines) or mimicking tRNA (puromycin). Furthermore, the idea of creating a programmable RNA-antibiotic able to recognize and inhibit in a sequence-specific manner bacterial RNAs began to be pursued during the 80s in the workhorse model organism *E. coli*, implementing short oligonucleotides targeting rRNA [84]. Following other pioneering studies in the same organism, the exciting approach of inhibiting transcripts of essential genes by using antisense short oligonucleotides (ASOs) has been explored in several Gram-positive and Gram-negative bacteria [85,86]. According to the same principle followed for targeting essential genes with antisense RNAs, the idea of targeting regulatory sRNA with crucial functions in virulence and antibiotic resistance is extremely appealing and, in our opinion, has to be pursued. The use of RNA molecules as antibacterial compounds has the advantages of being highly specific and of requiring a simple chemical synthesis. However, considering the instability of the RNA molecule in its unmodified state, several classes of modified ASOs have been developed with increased stability and resistance to nuclease attack. Specifically, locked nucleic acids (LNA), phosphorodiamidate morpholino oligomers (PMO), and peptide nucleic acids (PNA) nowadays represent the available ASOs, with the latter two being the most popular as an antibacterial molecule [87]. While LNA and PMO are modified nucleic acids, PNA is a synthetic polymer, highly stable and resistant to protease and nuclease digestion, with a pseudo-peptide backbone and attached nucleobases [88]. Due to the limited uptake of nucleic acids by bacterial cells, ASOs are linked to short bacterial penetrating peptides (BPP), which are constituted by less than 30 amino acids and are amphiphilic or cationic. The attachment of these peptide tags significantly improves ASOs delivery inside the cell. Considering the plethora of mechanisms adopted by regulatory sRNAs, peptide-conjugated ASOs could act in principle in different ways, depending on the mode of action of the targeted sRNA (Figure 2). The most obvious scenario implies that ASO interaction with a sRNA would inhibit sRNA base-pairing, preventing the regulatory outcome triggered by the riboregulator. In theory, the sequestration of the sRNA by base-pairing ASO could be accompanied by other events affecting sRNA stability. For example, upon sRNA-ASO interaction, the regulatory RNA could go through a structural rearrangement, affecting sRNA ability to interact with its partner RNA chaperone, and ultimately, its stability and its function. In addition, the formation of a sRNA-ASO duplex could render the regulatory RNA more or less prone to active degradation by cellular RNase enzymes. Considering the lack of detailed studies in this field, the effects of sequence-specific sRNA targeting with specific ASOs can be nowadays only hypothesized, but this strategy may represent a new way for treating antimicrobial-resistant pathogens.

## 4. Concluding Remarks

During the last century, the use of antibiotics has represented the pillars of modern medicine to fight bacterial infections. On the other hand, the use of antibiotics has contributed to the onset and spread of pathogenic strains resistant to the treatment. As a result, it is becoming increasingly difficult to treat bacterial infections successfully, and more efforts must address this emergency. To this end, it would be desirable that governments and the academic and industrial scientific communities focus more resources and actions on discovering new antibacterial drugs. This perspective article highlighted the high potential of bacterial transcriptional regulators as targets for developing new drugs. These compounds should be capable of binding and specifically blocking the functioning of transcriptional regulators necessary to express genes involved in bacterial pathogenesis. At the same time, they should not target host proteins and be non-toxic for the organism. To this purpose, some compounds were screened among FDA-approved non-toxic chemicals in a drug repurposing approach [20,21,43]. For the new molecules, many of them proved to be non-toxic for mammalian cells [13,19,22,23,24] and rodents [16,26,39,45,47,51], while many other compounds were toxic for the host and had to be excluded from further studies [22]. It is also becoming more evident and convincing that this goal could be achieved using sRNAs that can specifically interfere with the expression of genes important for bacterial pathogenesis through base pairings with their targets.

These fields of investigation would be effective and efficient with greater coordination between groups with complementary skills and technologies. For example, biochemistry, molecular biology, and genetics skills should integrate even more with bioinformatics skills and high throughput screening approaches to predict and identify new molecules capable of blocking the regulatory activity of DNA-binding TRs and base-pairing sRNAs.

## Figures and Tables

**Table 1:** List of target TRs responding to inhibitory compounds in several microorganisms.

Target TR	Category of Targeted TR	Microorganism	Inhibitory Effects of the Compounds on the TR	Effects of the Compounds on the Bacterium (Type of Test, Amount of Compound)	Refs.
**PrfA**	Activator	*L. monocytogenes*	DNA-binding	Reduce virulence (DNA-binding IC_50_ 6–7 μM)	[11]
**PrrB/PrrA**	HK	*M. tuberculosis*	Unknown	Bactericidal (MIC 0.06–8 μg/mL)	[12]
**DosT/DosS/DosR**	HKs, RR	*M. tuberculosis*	Sensor of the HKs, autophosphorylation of HK, or DNA-binding of the RR	Decrease the survival in specific growth conditions (EC_50_ 0.6–9.8 μM)	[13,14]
**PhoR/PhoP**	RR	*M. tuberculosis*	DNA-binding of the RR	Decrease virulence (IC_50_ 5 μM, 100 μM in macrophages, 100 mg/Kg in mice)	[15,16]
**PhoQ/PhoP**	HK, RR	*S. enterica*	Autophosphorylation of HK or DNA-binding of the RR	Unknown (DNA-binding IC_50_ 3.6–285 μM)	[17,18]
**SsrA/SsrB**	RR	*S. enterica*	Unknown	Decrease virulence and sensitize the pathogen to other antibiotics (2.5 mg/Kg in mice)	[19]
**PmrB/PmrA**	RR	*S. enterica*	Unknown	Decrease virulence and sensitize the pathogen to other antibiotics (2.5 mg/Kg in mice)	[19]
**HsrA (HP1043)**	Orphan RR	*H. pylori*	DNA-binding	Bactericidal (MIC/MBC 4–128 mg/L)	[20]
**ArsS/ArsR**	RR	*H. pylori*	DNA-binding	Bactericidal (MIC/MBC 32–128 mg/L)	[21]
**VirF**	Activator	*S. flexneri*	Unknown	Decrease virulence and host invasion (IC_50_ 14–66 μM)	[22]
**PhoQ/PhoP**	HK	*S. flexneri*	Autophosphorylation of HK	Decrease virulence and host invasion (enzymatic inhibition IC_50_ 8–70 μM)	[23]
**ToxT**	Activator	*V. cholerae*	Dimerization or ToxT expression	Decrease host colonization (IC_50_ 20–30 μM, EC_50_ 2.7–25 μM, 100–200 mg/mouse)	[24,25,26]
**VanS/VanR**	RR	*E. faecium*	Phosphoryl transfer to RR or DNA-binding of the RR	Unknown (DNA-binding IC_50_ 3 μM)	[27,28]
**WalK/WalR**	HK, RR	*S. pneumoniae* *S. pyogenes* *S. epidermidis* *S. mutans* *E. faecalis* *B. subtilis* *S. aureus*	Dimerization of HK, autophosphorylation of HK, phosphoryl transfer to RR or dimerization of the RR	Reduce bacterial growth (enzymatic inhibition IC_50_ 37–62 μM, MIC 0.39–128 μg/mL or 8–16 μM)	[29,30,31,32]
**CiaH/CiaR**	HK	*S. mutans*	Autophosphorylation of HK	Decrease virulence (enzymatic inhibition IC_50_ 4.9 μM, in vivo 0.63 μg/mL)	[33]
**VicK/VicR**	HK	*S. mutans*	Autophosphorylation of HK	Decrease virulence (enzymatic inhibition IC_50_ 2.9 μM, in vivo 0.63 μg/mL)	[33]
**LiaS/LiaR**	HK	*S. mutans*	Autophosphorylation of HK	Decrease virulence (enzymatic inhibition IC_50_ 5.6 μM, in vivo 0.63 μg/mL)	[33]
**HpkA/DrrA**	HK	*T. maritima*	Autophosphorylation of HK	Unknown (enzymatic inhibition IC_50_ 0.4–2.3 μM)	[34]
**Fur**	Activator and repressor	*E. coli*	Dimerization and/or DNA-binding	Decrease virulence (ND)	[35]
**EnvZ/OmpR**	HK	*E. coli*	Autophosphorylation of HK	Unknown (enzymatic inhibition IC_50_ 1.2 μM)	[33]
**PhoP/PhoQ**	HK	*E. coli*	Autophosphorylation of HK	Unknown (enzymatic inhibition IC_50_ 1.2 μM)	[33]
**CheA/CheY**	HK	*E. coli*	Autophosphorylation of HK	Unknown (enzymatic inhibition at 50 μg/mL)	[36]
**NtrB/NtrC**	HK	*E. coli*	Autophosphorylation of HK	Unknown (enzymatic inhibition at 50 μg/mL)	[36]
**BasS/BasR**	RR	*E. coli*	Phosphoryl transfer to RR	Reduce growth in specific conditions (growth inhibition at 50 μg/mL)	[37]
**CreC/CreB**	RR	*E. coli*	Phosphoryl transfer to RR	Reduce growth in specific conditions (growth inhibition at 50 μg/mL)	[37]
**KinA/SpoOF**	HK	*B. subtilis*	Autophosphorylation of HK	Unknown (enzymatic inhibition at 50 μg/mL)	[36,38]
**AlgR2/AlgR1**	HK, RR	*P. aeruginosa*	Autophosphorylation and dephosphorylation of HK, phosphoryl transfer to RR, or DNA-binding	Unknown (enzymatic inhibition at 50 μg/mL)	[36]
**PqsR**	Activator	*P. aeruginosa*	Signal sensing	Reduce virulence factor production and biofilm formation (IC_50_ 0.2–39 μM)	[39,40,41,42,43]
**QseC/QseB** **QseC/QseF** **QseC/KdpE**	HK	Many Gram-negative bacteria	Autophosphorylation of HK	Decrease virulence (20 mg/Kg in mice)	[44,45]
**AgrC/AgrA**	HK, RR	*S. aureus*	Signal sensing by HK or DNA-binding	Decrease virulence (IC_50_ 10–90 nM, DNA-binding IC_50_ 83 μM)	[46,47]
**AgrC/AgrA**	HK	*S. epidermidis*	Signal sensing by HK	Decrease virulence (IC_50_ 2–50 nM)	[48]
**ComD/ComE**	HK	*S. pneumoniae*	Signal sensing by HK	Decrease virulence and horizontal gene transfer (IC_50_ 86–670 nM, EC_50_ 6–83 nM)	[49,50]
**FsrC/FsrA**	HK	*E. faecalis*	Signal sensing by HK	Reduce host invasion and virulence (IC_50_ 0.026–5 μM)	[51]
**BfmS/BfmR**	RR	*A. baumannii*	Unknown	Reduce biofilm formation (IC_50_ 10 μM)	[52]

Symbols: TR, transcription regulator; HK, histidine kinase; RR, response regulator; IC_50_, half maximal inhibitory concentration; EC_50_, half maximal effective concentration; MIC, minimum inhibitory concentration; MBC, minimum bactericidal concentration; ND, not determined; Underlined regulators refer to targeted proteins.

## Data Availability

Not applicable.
